# The effect of heparin infusion intensity on outcomes for bridging hospitalized patients with atrial fibrillation

**DOI:** 10.1002/clc.23256

**Published:** 2019-09-04

**Authors:** Bruce A. Warden, Calvin Diep, Ran Ran, Matthew Thomas, Joaquin E. Cigarroa

**Affiliations:** ^1^ Department of Pharmacy Services Oregon Health & Science University Portland Oregon; ^2^ Department of Pharmacy Stanford Medical Center Stanford California; ^3^ Department of Critical Care Medicine Oregon Health & Science University Portland Oregon; ^4^ Department of Pharmacotherapy Washington State University College of Pharmacy and Pharmaceutical Sciences Spokane Washington; ^5^ Division of Cardiology, Knight Cardiovascular Institute Oregon Health & Science University Portland Oregon

**Keywords:** anticoagulation, atrial fibrillation, bleeding, bridging, heparin, thrombosis

## Abstract

**Background:**

Perioperative bridging in atrial fibrillation (AF) is associated with low thromboembolic rates but high bleeding rates. Recent guidance cautions the practice of bridging except in high risk patients. However, the practice of bridging varies widely and little data exist regarding appropriate anticoagulation intensity when using intravenous unfractionated heparin (UFH).

**Hypothesis:**

To determine if high intensity UFH infusion regimens are associated with increased bleeding rates compared to low intensity regimens for bridging patients with AF.

**Methods:**

We conducted a single center retrospective cohort study of admitted patients with non‐valvular AF receiving UFH for ≥24 hours. UFH intensities were chosen at the providers' discretion. The primary endpoint was the rate of bleeding defined by the International Society on Thrombosis and Hemostasis during UFH infusion or within 24 hours of discontinuation. The secondary endpoint was a composite of cardiovascular events, arterial thromboembolism, venous thromboembolism, myocardial infarctions and death during UFH infusion.

**Results:**

A total of 497 patients were included in this analysis. Warfarin was used in 82.1% and direct acting oral anticoagulants in 14.1% of patients. The rate of any bleed was higher among high intensity compared to low intensity UFH regimens (10.5% vs 4.9%, odds ratio = 2.29, 95% confidence interval = 1.07‐4.90). Major bleeding was significantly higher among high intensity compared to low intensity UFH regimens. There was no difference in composite thrombotic events or death.

**Conclusions:**

Low intensity UFH infusions, targeting lower anticoagulation targets, were associated with decreased bleeding rates without a signal of increased thromboembolic events in hospitalized AF patients.

## 1 INTRODUCTION

Patients with atrial fibrillation (AF) have a 4‐ to 5‐fold increased risk of ischemic stroke compared to patients in sinus rhythm.^1^ The annual risk of stroke is 1.69% to 4.2% in patients with AF, with 15% of strokes in the United States attributed to AF.^2^ To mitigate this risk, hospitalized patients are often treated with parenteral anticoagulation during perioperative interruption of oral anticoagulation or while oral anticoagulation is sub‐therapeutic.

Recent studies suggest perioperative bridging in patients with AF provides limited benefit in terms of preventing thromboembolic events, but increases bleeding rates.^3^, ^4^, ^5^, ^6^, ^7^ Most studies, including the BRIDGE trial,^3^ the only randomized placebo‐controlled trial, evaluated perioperative bridging strategies for elective surgeries, whereas the data for bridging hospitalized patients with acute issues has until recently not been addressed.^5^ These studies enrolled predominantly low to moderate thromboembolic risk patients (mean CHADS_2_ = 2.1‐2.4 and CHA_2_DS_2_‐VASc = 3.6‐4.1) and limited patients at higher thromboembolic risk. Studies have also shown that perioperative bridging in AF is associated with a 4‐fold risk of bleeding compared to non‐bridging strategies (any bleed rates: 5%‐34% and major bleed rates: 3%‐9%) with undifferentiated thromboembolic events (0%‐4%).^3^, ^4^, ^5^, ^6^, ^7^ Current guidelines and expert consensus support perioperative bridging with subcutaneous low molecular weight heparin or intravenous unfractionated heparin (UFH) for patients taking oral vitamin K antagonists who are at high risk for thromboembolism, indicated by a CHA_2_DS_2_‐VASc score greater than 5 to 6 or a prior thromboembolic event.^8^, ^9^, ^10^, ^11^, ^12^, ^13^, ^14^ However, there is currently limited data and differing consensus within guidelines and literature regarding UFH dosing intensity for bridging in AF. The perioperative antithrombotic CHEST guidelines recommend targeting an activated partial thromboplastin time (aPTT) of 1.5 to 2.0 times the control aPTT value (considered low intensity), while the antithrombotic therapy in AF CHEST guidelines recommend targeting an anti‐Xa of 0.3 to 0.7 units/mL (considered high intensity).^10^, ^11^ Furthermore, many major guidelines do not provide any recommendations on anticoagulation targets for bridging in AF.^8^, ^13^, ^14^ Comparing the safety and efficacy of high vs low intensity UFH infusion regimens has not been addressed.

This study is designed to investigate bleeding rates in hospitalized patients with non‐valvular AF receiving high or low intensity UFH infusions for bridging. The results of this study will provide guidance on UFH infusion intensity and anticoagulation targets to minimize bleeding risks in this patient population.

## 2 METHODS

This is a single center retrospective cohort study of patients admitted to Oregon Health & Science University (OHSU), a 576‐bed academic medical center in Portland, Oregon, from 1 April 2008 to 1 September 2016. Inclusion criteria included age ≥18 years, receiving UFH for >24 hours for anticoagulant bridging, a known diagnosis of AF in the absence of rheumatic mitral stenosis, a mechanical or bioprosthetic heart valve, or mitral valve repair.^13^ Patients were excluded if they were not on chronic oral anticoagulation therapy or if they were on therapeutic anticoagulation for indications other than AF (ie, venous thromboembolism, arterial thromboembolism, ventricular assist devices, etc.). This study was approved by the OHSU institutional review board.

The Crystal reporting system and EPIC electronic medical record was used to identify hospitalized patients with AF receiving intravenous UFH. Patient screening for inclusion and data collection was obtained through retrospective chart review by three individual investigators. The following information was collected for patients that met pre‐specified inclusion criteria: baseline demographics, UFH protocol used, use of boluses, bolus doses and documented bleeding, cardiovascular or mortality events. CHA_2_DS_2_‐VASc scores, HAS‐BLED scores, time on UFH, average UFH dose, and percent time in therapeutic range based on selected UFH protocol were calculated for each patient included.

UFH regimens were chosen at the discretion of the treating physicians as there is no recommended dose regimen for bridging with UFH in AF. UFH intensity was defined based on target aPTT or anti‐Xa levels within specified UFH infusion protocols, see Table S1. High intensity regimens targeted an aPTT of 76 to 120 seconds or anti‐Xa levels of 0.35 to 0.7 units/mL. Low intensity regimens targeted an aPTT of 46 to 70 seconds or anti‐Xa level of 0.35 to 0.5 units/mL, see Table S2. Patients receiving multiple UFH infusion regimens during their hospitalization were documented as receiving the highest intensity regimen lasting ≥24 hours.

The primary outcome was rate of any bleeding, defined as a documented bleeding event during UFH infusion or within 24 hours of UFH discontinuation. In accordance with previous studies of perioperative bridging in patients with AF, our study classified major and minor bleeding as defined by the International Society of Thrombosis and Hemostasis criteria, Table S3.^15^, ^16^ The secondary outcome was a composite of cardiovascular events, which included arterial thromboembolism (stroke or systemic embolism), venous thromboembolism (deep vein thrombosis or pulmonary embolism) or myocardial infarction defined as an event that occurred during UFH infusion not attributed to other causes. Differences between high and low intensity UFH regimens for the individual components of the composite cardiovascular events as well as rate of death were also assessed. All bleeding and cardiovascular events were reviewed by two individual investigators.

Published rates of bleeding in bridged patients are widely variable owing to different patient populations and different bridging methods. Of the studies reviewed, Roswell et al^5^ bore the most similar design, evaluating bleeding incidence for inpatient UFH bridging. The sample size for our study was based on the bleed rates published in Roswell et al which showed a 34% rate for any bleed and 9% rate for major bleeds for patients bridged with UFH infusion. Since there were no previous studies addressing the rate of bleeding for lower intensity UFH infusions, we proposed that an absolute reduction of any bleed event by 12% would be clinically significant. A sample size of 432 patients would be required to have an 80% chance of detecting this difference at a significance of 5%.

All continuous variables were tested for normality by Kolmogorov‐Smirnov statistic and Shapiro‐Wilk statistic. Nonparametric continuous variables were analyzed using Mann‐Whitney *U* test, and categorical variables were compared with Pearson chi‐squared test, Fisher exact test, or likelihood ratio test. A subgroup analysis was performed on patients with CHA_2_DS_2_‐VASc scores ≥5 comparing rates of bleeding and complications. Potential imbalances in the demographic variables were to be adjusted with logistic regression and potential imbalances in duration of heparin therapy were to be adjusted with log‐rank test.

## 3 RESULTS

**Figure 1:**
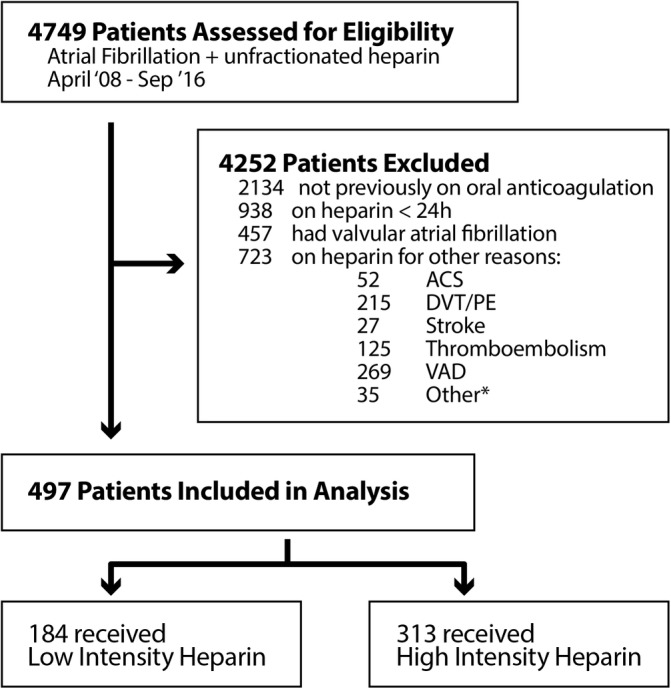
Patient cohort for primary analysis including the total number of hospitalized patients with AF on intravenous UFH infusion and reasons for exclusion. ACS, acute coronary syndrome; DVT/PE, deep vein thrombosis/pulmonary embolism; VAD, ventricular assist device. *Other: aquapheresis, congenital heart disease, carotid stent, continuous renal replacement therapy, hypercoagulable state, procedural, and heart transplant

From 1 April 2008 to 1 September 2016, a total of 4749 hospitalized patients were identified as having AF and receiving intravenous UFH during their admission. Of these patients, 497 were included for analysis (Figure 1). Baseline characteristics are shown in Table 1. There were no statistically significant differences in any of the baseline characteristic among the groups. The median age was 66 years, 31.8% were female and the median body weight was 87.1 kg. The median CHA_2_DS_2_‐VASc score was 4 with 31.4% of the patients having a CHA_2_DS_2_‐VASc score of 5 or greater. The median HAS‐BLED score was 4. Other medications noted at baseline: 65.8% of patients were taking aspirin, 40.2% were taking a non‐steroidal anti‐inflammatory drug, 64.6% were taking an antiplatelet, 12.7% were on dual antiplatelets, 82.1% were taking warfarin and 14.1% were taking a direct acting oral anticoagulant.

**Table 1:** Baseline characteristics

Characteristics	Low intensity (n = 184)	High intensity (n = 313)	*P*‐value
Age, median (Q2, Q3)	68 (58‐76)	66 (56‐75)	.297
Female, no. (%)	50 (27.2)	108 (34.5)	
Weight (kg), median (Q2, Q3)	87.1 (75.1‐103.4)	86.8 (69.6‐107.3)	.611
Comorbidities			
Chronic heart failure	98 (53.5)	157 (50.2)	.504
Hypertension	120 (65.2)	205 (65.5)	.950
Diabetes	60 (32.6)	123 (39.3)	.135
Prior stroke/TIA/thromboembolism event	45 (24.5)	80 (25.6)	.784
Prior vascular disease	94 (51.1)	155 (49.5)	.736
History of renal disease	54 (29.3)	82 (26.2)	.447
History of liver disease	42 (23.4)	76 (24.3)	.713
Prior bleed	20 (10.9)	43 (13.7)	.353
CHA_2_DS_2_‐VASc			
Mean	3.66	3.7	.587
Median	3	4	
CHA_2_DS_2_‐VASc ≥5, no. (%)	52 (28.3)	104 (33.2)	
Distribution, no. (%)			
0	2 (1.1)	11 (3.5)	
1	23 (12.5)	23 (7.3)	
2	31 (16.8)	57 (18.2)	
3	40 (21.7)	65 (20.8)	
4	36 (19.6)	53 (16.9)	
5	17 (9.2)	39 (12.5)	
6	14 (7.6)	42 (13.4)	
7	15 (8.2)	17 (5.4)	
8	4 (2.2)	5 (1.6)	
9	2 (1.1)	1 (0.3)	
HAS‐BLED			
Mean	3.85	3.67	.190
Median	4	4	
Distribution, no. (%)			
1	6 (3.3)	23 (7.3)	
2	25 (13.6)	38 (12.1)	
3	38 (20.7)	76 (24.3)	
4	60 (32.6)	94 (30.0)	
5	38 (20.7)	52 (16.6)	
6	12 (6.5)	23 (7.3)	
7	4 (2.2)	7 (2.2)	
8	1 (0.5)	0 (0.0)	
Medications use, no. (%)			
NSAID	74 (40.2)	140 (44.7)	.327
Aspirin	130 (70.7)	197 (62.9)	.080
Clopidogrel	31 (16.8)	33 (10.5)	.059
Antiplatelet^a^	121 (71.2)	200 (63.9)	.104
Dual antiplatelet therapy^b^	30 (16.3)	33 (10.5)	.084
Warfarin	155 (84.2)	253 (80.8)	.717
DOAC	25 (13.6)	45 (14.3)	.717

Abbreviations: DOAC, direct acting oral anticoagulant; no., number; NSAID, non‐steroidal anti‐inflammatory; Q2, quartile 2; Q3, quartile 3.

a Aspirin, clopidogrel or dipyradimole.

b Aspirin and clopidogrel.

The median duration of UFH was 86 and 83 hours in patients who received high and low intensity UFH regimens, respectively, *P* = .69. Duration of hospitalization was a median of 12.1 days in the high, and 11.6 days in the low intensity UFH regimens, respectively, *P* = .19. During this time, 76% and 76.6% of patients in the high and low intensity UFH regimens, respectively, underwent a procedure, *P* = .971. There was no difference in the classification (non‐surgical vs surgical) or individual type of procedures.

**Figure 2:**
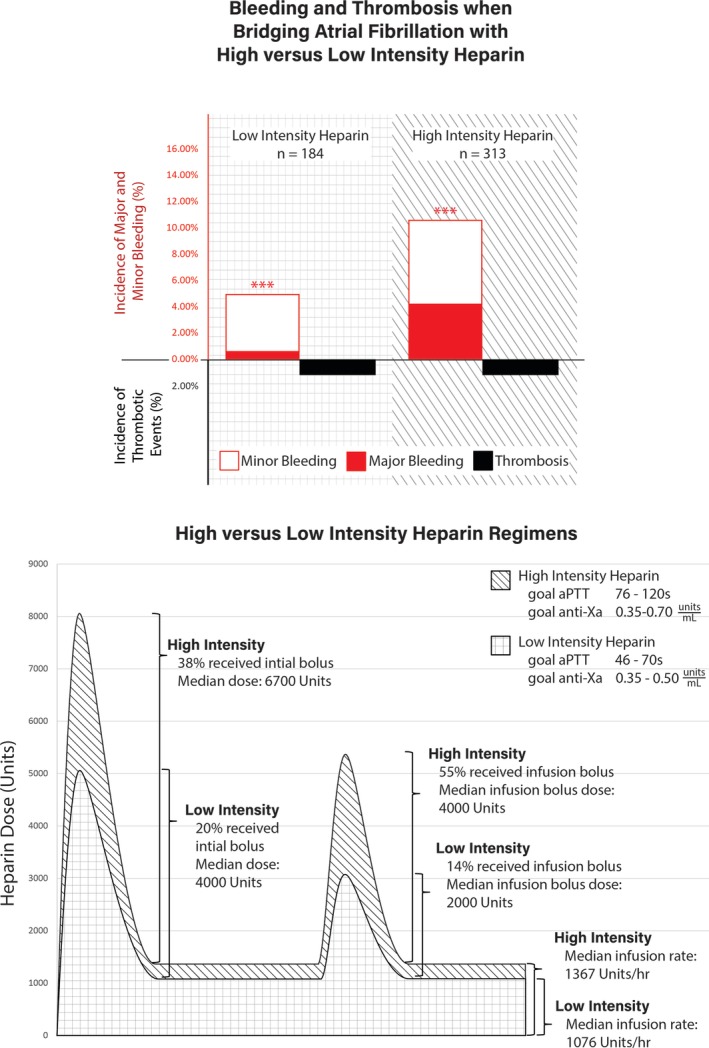
Outcome (top) and pharmacokinetic (bottom) differences among high intensity vs low intensity heparin regimens. In this study, high compared to low intensity heparin regimens was associated with greater usage of bolus doses and higher infusion rates, corresponding to a significantly increased occurrence of both minor and major bleeding events without reducing thromboemboli

The median percent time in therapeutic range was 50% without significant differences between the two groups. The overall median UFH bolus doses (initial bolus: 6700 units vs 4000 units, *P* < .01; infusion bolus: 4000 units vs 2000 units, *P* < .01) and infusion rate (15 units/kg/h vs 12 units/kg/h, *P* < .01; 1367 units/h vs 1076 units/h, *P* < .01) were significantly higher in patients who received high vs low intensity regimens. A higher proportion of patients received initial UFH boluses in the high intensity compared to low intensity UFH group (38.3% vs 19.6%, *P* < .01). Patients receiving high intensity UFH regimens were also more likely to receive boluses during maintenance UFH infusions (54.6% vs 14.1%, *P* < .01). Overall, patients in the high intensity group had a greater total exposure to UFH than did the lower intensity group (Figure 2). All UFH parameters are shown in Table 2.

**Table 2:** Heparin parameters

Parameters	Low intensity (n = 184)	High intensity (n = 313)	*P*‐value
Initial bolus given, no. (%)	36 (19.6)	120 (38.3)	<.01
Initial bolus dose (units), median (Q2, Q3)	4000 (4000‐4801)	6700 (4650‐8000)	<.01
Heparin dose (units/h), median (Q2, Q3)	1076 (900‐1327)	1367 (1092‐1680)	<.01
Heparin dose (units/kg/h), median (Q2, Q3)	12 (10‐14)	15 (13‐18)	<.01
Infusion bolus given, no. (%)	26 (14.1)	171 (54.6)	<.01
Infusion bolus dose, median (Q2, Q3)	2000 (2000‐3750)	4000 (3250‐5200)	<.01
Time on heparin (h), median (Q2, Q3)	83 (54‐127)	86 (49‐133)	.69
Percentage time in therapeutic range, median (Q2, Q3)	50 (33‐67)	50 (33‐66)	.70

Abbreviations: no., number; Q2, quartile 2; Q3, quartile 3.

The rate of any bleeding event was significantly higher in patients who received high vs low intensity UFH regimens (10.5% vs 4.9%, odds ratio [OR] = 2.29, 95% confidence interval [CI] = 1.07‐4.90). Major bleeding occurred in 4.2% and 0.5% of patients who received high and low intensity UFH regimens, respectively (OR = 7.93, 95% CI = 1.03‐61.1) (Figure 2). Minor bleeding occurred in 6.4% and 4.3% of patients who received high and low intensity UFH regimens, respectively (OR = 1.50, 95% CI = 0.65‐3.48). Approximately, 74% of patients who experienced a bleeding event were on concomitant warfarin therapy. The average international ratio at the time of documented bleed was similar between the two groups: 1.49 in patients receiving high intensity group and 1.59 in patients receiving low intensity group (*P* = .41). Information regarding each bleeding event is provided in Table S4.

The rate of secondary endpoints of composite cardiovascular events, stroke, myocardial infarction, arterial thromboembolism, venous thromboembolism and death was low and not significantly different in patients receiving high and low intensity UFH infusions (Figure 2). The rates of primary and secondary endpoints are shown in Table 3.

**Table 3:** Primary and secondary endpoints

	Low intensity (n = 184)	High intensity (n = 313)	Odds ratio (95% confidence interval)	*P*‐value
Primary endpoint				
Bleed,^a^ no. (%)	9 (4.9)	33 (10.5)	2.29 (1.07‐4.90)	.029
Major	1 (0.5)	13 (4.2)	7.93 (1.03–61.1)	.022
Minor	8 (4.3)	20 (6.4)	1.50 (0.65–3.48)	.34
Secondary endpoint				
Composite cardiovascular events	2 (1.1)	4 (1.3)	0.85 (0.15‐4.69)	1.00
Arterial thromboembolism, no. (%)	2 (1.1)	2 (0.6)	0.56 (0.08‐4.19)	.62
Stroke	2 (1.1)	0 (0.0)	N/A	.14
Systemic embolism	0 (0.0)	2 (0.6)	N/A	.53
Venous thromboembolism	0 (0.0)	2 (0.6)	N/A	.53
Myocardial infarction	0 (0.0)	0 (0.0)	N/A	N/A
Death	0 (0.0)	2 (0.6)	N/A	.53

Abbreviation: no., number; N/A, not applicable.

a Major and minor bleeds defined per International Society on Thrombosis and Hemostasis criteria.

In patients with a CHA_2_DS_2_‐VASc score >5, there was a trend towards increased bleeding events in patients who received high intensity UFH regimens compared to those who received low intensity UFH regimens (10.6% vs 1.9%, *P* = .11). Thromboembolic events were also low in patients with CHA_2_DS_2_‐VASc scores >5 with no significant difference between patients who received high vs low intensity UFH regimens.

## 4 DISCUSSION

In this single center retrospective cohort study evaluating 497 hospitalized patients with non‐valvular AF receiving parenteral bridge therapy with UFH, a higher intensity UFH regimen (aPTT: 76‐120 seconds, anti‐Xa level: 0.35‐0.7 units/mL) as opposed to a lower intensity UFH regimen (aPTT: 46‐70 seconds, anti‐Xa level: 0.35‐0.5 units/mL) resulted in significantly increased rates of the primary endpoint of any bleeding (10.5% vs 4.9%, OR = 2.29, 95% CI = 1.07‐4.90) and major bleeding (4.2% vs 0.5%, OR = 7.93, 95% CI = 1.03‐61.1), without reducing the risk of thromboemboli. This is the first study to compare different UFH targets for bridging in AF.

Two deaths were observed in this study. Both occurred in patients who received high intensity UFH regimens during their admission. However, UFH could not be definitively defined as the cause of death in either patient given the complexity of the clinical scenario, procedures and extensive comorbidities.

It is well established that bleeding occurs at a much higher rate than thromboembolism in patients who receive perioperative bridging, at an approximate bleed to thrombosis ratio of 13:1 with a marked increase in the risk of bleeding (OR = 3.6, 95% CI = 1.52‐8.50).^17^ Prior studies in the low to moderate risk AF population show increased bleeding events and similar thromboembolic events in those who received perioperative bridging compared to those who did not.^3^, ^4^, ^5^, ^6^, ^7^ In 2017, an expert consensus recommended the use of parenteral anticoagulation for bridging patients with AF only in patients with a CHA_2_DS_2_‐VASc score ≥5 and/or a prior thromboembolic event.^8^ Furthermore, the 2019 AHA/ACC/HRS focused update on the guideline for management of AF recommends balancing the risk of stroke and bleeding when deciding if a bridging strategy should be implemented.^13^ However, the evidence for bridging remains scarce as no studies have shown a decrease in thromboembolic events with a bridging strategy in low to moderate risk patients, and high risk patients have not been adequately studied in this setting. Defining optimal anticoagulation strategies and therapeutic targets are essential to minimize the bleeding risk without increasing systemic thromboemboli.

A recent study demonstrated the risk of excess bleeding with higher initial UFH infusion rates in patients who received UFH for bridging in AF.^5^ The Roswell et al study demonstrated that the median initial UFH rate was higher in those who experienced bleeding events compared to those without bleeding events (13.3 units/kg/h vs 11.4 units/kg/h, *P* = .012).^5^ Our study aimed to further characterize this observation by comparing two distinct anticoagulation intensities (high vs low) for bridging patients with AF. In our study, the median UFH infusion rate was significantly higher in patients receiving high intensity regimens compared to low intensity regimens, 15 units/kg/h vs 12 units/kg/h, respectively, which was associated with increased any and major bleeding events.

The present study has implications for both clinical practice and future clinical studies in hospitalized patients with AF requiring parenteral bridge therapy. Our findings, for the first time, demonstrated a reduction in major bleeding without a signal of excess systemic thromboemboli in hospitalized AF patients treated with a low intensity as opposed to a high intensity UFH bridging strategy. In our study, the use of high intensity UFH increased the odds of bleeding 2‐fold as compared to low intensity UFH infusions (OR = 2.3, 95% CI = 1.1‐4.9), without a change in rate of systemic thromboemboli. Adoption of a low intensity UFH regimen in hospitalized AF patients requiring bridging would reduce any bleeding in approximately 6400 patients and major bleeding in approximately 4200 patients per year in the United States. This is based on an extrapolation from 326 000 000 million Americans,^18^ 2115 AF hospitalizations per 1 million US population per year (assuming a 14.4% relative increase from 2010 statistics),^19^ 48% of AF patients being anticoagulated with 64% of those being bridged and 54% of them using UFH,^20^ equating to 114 411 AF patients being bridged with UFH per year. Applying the results of this study, a 5.6% absolute reduction in any bleed and 3.7% absolute reduction in major bleeds, would estimate the bleed events averted as noted above. Given the large and growing population of patients with AF, the frequency at which bridge therapy is required and the associated bleeding risk, strategies to minimize adverse outcomes remains a public health priority. Therefore, large multicenter prospective studies are needed to validate these findings, as well as investigations into the optimal UFH infusion dosing intensity for bridging in AF patients with the highest thromboembolic risk.

Limitations of this study include the single center and retrospective nature and potential confounders inherent with such a design. Measures taken to limit confounding include a well‐balanced and similar baseline patient population and the exclusion of patients on intravenous UFH for non‐AF conditions as their primary indication for bridging. UFH protocols and medical practice may vary among institutions. Differences in physician, pharmacy and nursing practice can affect the degree of UFH infusion prescribing, dosing, titration, and monitoring. Studies have suggested that monitoring UFH infusions with anti‐Xa levels compared to aPTT is superior in maintaining values within goal range.^21^, ^22^ However, therapeutic dosing of UFH with regards to safety and efficacy has been guided by aPTT, not anti‐Xa levels in clinical trials, mostly acute coronary syndrome. This study did not assess the difference in bleeding events and the type of monitoring used (aPTT or anti‐Xa). The type of monitoring was chosen at the discretion of the providers and the baseline characteristics were well balanced between the two groups. In addition, as can be seen in the heparin protocols viewed in Tables S1 and S2, there were some overlap in anticoagulation intensities as measured by therapeutic goal ranges (ie, mechanic valve protocol [high intensity] and heart failure protocol [low intensity] have a goal anti‐Xa levels of 0.35‐0.7 and 0.35‐0.5 units/mL, respectively). The small sample size limited the ability to detect differences in the secondary endpoints. However, prior studies were also unable to detect differences in thromboembolic events given the inherent low rate of occurrence in bridging and large numbers needed to show such a small difference.^3^, ^4^, ^5^, ^6^, ^7^

The results of this study demonstrate that use of a low intensity UFH regimen in hospitalized AF patients can decrease major morbidity by decreasing both any and major bleeding event rates without a signal of increasing systemic thromboemboli. Low intensity UFH should now be the preferred dosing strategy for this population of patients in clinical practice.

## CONFLICT OF INTEREST

The authors declare no potential conflict of interests.

## Supporting information


**Table S1**. OHSU heparin protocols.

**Table S2**. Heparin dosing protocols.

**Table S3**. International Society on Thrombosis and Hemostasis (ISTH) bleeding definition.

**Table S4**. Bleeding events.
