# Supplementary material for: The effect of heparin infusion intensity on outcomes for bridging hospitalized patients with atrial fibrillation
Source: Clin Cardiol. 2019 Sep 4;42(10):995–1002. doi: 10.1002/clc.23256 (PMC6788575; doi:10.1002/clc.23256)
Supplement: Supplementary file 1 — Table S1. OHSU heparin protocols. [file CLC-42-995-s001.docx]

**Supplemental Material Online Table 1:** OHSU Heparin Protocols

| **High Intensity** | |
| --- | --- |
| Venous thromboembolism | Goal anti-Xa: 0.35-0.7 units/mL |
| Mechanical valve | Goal aPTT: 76-120 seconds |
| **Low Intensity** | |
| Acute coronary syndrome | Goal aPTT: 46-70 seconds |
| Stroke | Goal aPTT: 50-70 seconds |
| Heart failure | Goal anti-Xa: 0.35-0.5 units/mL |

aPTT = activated partial thromboplastin time
