# Supplementary material for: The effect of heparin infusion intensity on outcomes for bridging hospitalized patients with atrial fibrillation
Source: Clin Cardiol. 2019 Sep 4;42(10):995–1002. doi: 10.1002/clc.23256 (PMC6788575; doi:10.1002/clc.23256)
Supplement: Supplementary file 2 — Table S2. Heparin dosing protocols. [file CLC-42-995-s002.docx]

**Supplemental Material Online Table 2:** Heparin Dosing Protocols

| **High Intensity Protocols** | | |
| --- | --- | --- |
| ***Venous thromboembolism***  Initial Bolus: 100 units/kg (age <60), 80 units/kg (age ≥60), max 10,000 units  Initial infusion rate: 18 units/kg/hr (age <60), 15 units/kg/hr (age ≥60), max 2000 units/hr | | |
| Anti-Xa value (units/mL) | Bolus | Rate change |
| <0.1 | 80 units/kg (max 10,000 units) | Increase by 4 units/kg/hr |
| 0.1-0.21 | 40 units/kg (max 5,000 units) | Increase by 2 units/kg/hr |
| 0.22-0.34 | None | Increase by 1 units/kg/hr |
| 0.35-0.7 | None | None |
| 0.71-0.95 | None | Decrease by 1 units/kg/hr |
| 0.96-1.2 | None | Decrease by 2 units/kg/hr |
| >1.2 | None | Stop infusion for 60 minutes  Decrease by 3 units/kg/hr |
| ***Mechanical valve***  Initial Bolus: 100 units/kg (age <60), 80 units/kg (age ≥60), max 10,000 units  Initial infusion rate: 18 units/kg/hr (age <60), 15 units/kg/hr (age ≥60), max 2000 units/hr | | |
| aPTT (seconds) | Bolus | Rate change |
| 55 | 80 units/kg (max 10,000 units) | Increase by 4 units/kg/hr |
| 55-65 | 40 units/kg (max 5,000 units) | Increase by 2 units/kg/hr |
| 66-75 | None | Increase by 1 units/kg/hr |
| 76-120 | None | None |
| 121-140 | None | Stop infusion for 30 minutes  Decrease by 2 units/kg/hr |
| >140 | None | Stop infusion for 60 minutes  Decrease by 3 units/kg/hr |
| **Low Intensity Protocols** | | |
| ***Acute coronary syndrome***  Initial Bolus: 600 units/kg, max 4,000 units  Initial infusion rate: 12 units/kg/hr, max 1,000 units/hr | | |
| aPTT (seconds) | Bolus | Rate change |
| <35 | 2000 units | Increase by 2 units/kg/hr |
| 35-45 | None | Increase by 2 units/kg/hr |
| 46-70 | None | None |
| 71-80 | None | Decrease by 1 units/kg/hr |
| 81-90 | None | Stop infusion for 30 minutes  Decrease by 2 units/kg/hr |
| >90 | None | Stop infusion for 60 minutes  Decrease by 3 units/kg/hr |
| ***Stroke***  Initial Bolus: None  Initial infusion rate: 15 units/kg/hr, max 2,000 units/hr | | |
| aPTT (seconds) | Bolus | Rate change |
| <45 | None | Increase by 4 units/kg/hr |
| 46-49 | None | Increase by 2 units/kg/hr |
| 50-70 | None | None |
| 71-95 | None | Decrease by 2 units/kg/hr |
| 95-120 | None | Decrease by 3 units/kg/hr |
| >120 | None | Stop infusion for 60 minutes  Decrease by 4 units/kg/hr |
| ***Heart failure***  Initial Bolus: None  Initial infusion rate: 12 units/kg/hr, max 1,000 units/hr | | |
| Anti-Xa value (units/mL) | Bolus | Rate change |
| <0.35 | None | Increase by 2 units/kg/hr |
| 0.35-0.5 | None | None |
| 0.51-0.7 | None | Decrease by 1 units/kg/hr |
| 0.71-0.9 | None | Stop infusion for 30 minutes  Decrease by 2 units/kg/hr |
| >0.9 | None | Stop infusion for 60 minutes  Decrease by 3 units/kg/hr |

Kg = kilogram

Hr = hour

aPTT = activated partial thromboplastin time

Anticoagulation monitoring targets are checked every 6 hours until two consecutive therapeutic results are obtained then targets are checked every 24 hours thereafter.
