# Supplementary material for: The effect of heparin infusion intensity on outcomes for bridging hospitalized patients with atrial fibrillation
Source: Clin Cardiol. 2019 Sep 4;42(10):995–1002. doi: 10.1002/clc.23256 (PMC6788575; doi:10.1002/clc.23256)
Supplement: Supplementary file 3 — Table S3. International Society on Thrombosis and Hemostasis (ISTH) bleeding definition. [file CLC-42-995-s003.docx]

**Supplemental Material Online Table 3:** International Society on Thrombosis and Haemostasis (ISTH) Bleeding Definition

| Major Bleed | 1. Fatal bleeding, and/or 2. Symptomatic bleeding in a critical area or organ, such as intracranial, intra-spinal, intraocular, retroperitoneal, intra-articular or pericardial, or intramuscular with compartment syndrome, and/or 3. Bleeding causing a fall in hemoglobin level of 20 g L^-1^ (1.24 mmol L^-1^) or more, or leading to transfusion of two or more units of whole blood or red cells. |
| --- | --- |
| Minor Bleed | Any bleeding not defined as a major bleeding |

g L^-1^ = grams per liter

mmol L^-1^ = millimole per liter
