# Supplementary material for: The effect of heparin infusion intensity on outcomes for bridging hospitalized patients with atrial fibrillation
Source: Clin Cardiol. 2019 Sep 4;42(10):995–1002. doi: 10.1002/clc.23256 (PMC6788575; doi:10.1002/clc.23256)
Supplement: Supplementary file 4 — Table S4. Bleeding events. [file CLC-42-995-s004.docx]

**Supplemental Material Online Table 4**: Bleeding events

| Type of Bleed | Number |
| --- | --- |
| Alverolar hemorrhage | 1^*^ |
| Intra-abdominal bleed | 2 |
| Intracranial hemorrhage | 1 |
| Retroperitoneal | 1^*^ |
| Subconjunctival | 1 |
| Hematoma | 16 |
| Melena/Hematemesis | 4 |
| Hematuria | 4 |
| Surgical Site | 6 |
| Epistaxis | 4 |
| Unspecified | 2 |

^*^patient passed following event
